# Plasma IL-6/IL-10 Ratio and IL-8, LDH, and HBDH Level Predict the Severity and the Risk of Death in AIDS Patients with* Pneumocystis* Pneumonia

**DOI:** 10.1155/2016/1583951

**Published:** 2016-08-07

**Authors:** Jia Sun, Junwei Su, Yirui Xie, Michael T. Yin, Ying Huang, Lijun Xu, Qihui Zhou, Biao Zhu

**Affiliations:** ^1^State Key Laboratory for Diagnosis and Treatment of Infectious Diseases, Zhejiang, Hangzhou 310006, China; ^2^The First Affiliated Hospital, School of Medicine, Zhejiang University, Zhejiang, Hangzhou 310006, China; ^3^Collaborative Innovation Center for Diagnosis and Treatment of Infectious Diseases, Zhejiang, Hangzhou 310006, China; ^4^Division of Infectious Diseases, Columbia University Medical Center, New York, NY 10032, USA

## Abstract

*Objective*. To identify blood biomarkers to predict severity and mortality in AIDS PCP patients.* Methods*. Biomarkers including clinical parameters and plasma inflammatory cytokines were assessed in 32 HIV-infected patients with* Pneumocystis* pneumonia (PCP) at time of admission. Predictive value of the biomarkers for clinical severity and in-hospital mortality was evaluated by corresponding ROC curve.* Results*. Levels of CRP, WBC, LDH, HBDH, and Ferritin were significantly higher in the severe and nonsurvivor AIDS PCP patients. These important biochemical indicators have inverse correlation with oxygenation index, especially levels of LDH (*P* = 0.008, *R* ^2^ = 0.258), HBDH (*P* = 0.001, *R* ^2^ = 0.335), and Ferritin (*P* = 0.005, *R* ^2^ = 0.237). Plasma IL-8 and IL-6 levels were significantly higher in patients with PaO_2_/FiO_2_ ≤ 200 mmHg and nonsurvivors than in those with PaO_2_/FiO_2_ > 200 mmHg and survivors. Severe and nonsurvival groups showed higher ratio of mean IL-6/IL-10 level (1.78 ± 1.56, *P* < 0.001; 1.11 ± 0.72, *P* = 0.043), larger AUC (95% CI 0.781–1.000, *P* < 0.001; 95% CI 0.592–0.917, *P* = 0.043), and more significantly inverse correlation with the oxygenation index.* Conclusion*. Plasma IL-8, LDH, and HBDH levels and IL-6/IL-10 ratio could be helpful for early evaluation of the severity and predicting fatal outcomes in AIDS PCP patients.

## 1. Introduction

*Pneumocystis* pneumonia (PCP) is one of the most common opportunistic infections in HIV-infected patients with a substantial mortality rate. A recent cohort study in China by Xiao et al. found that the top three most common opportunistic infections (OI) of hospitalized HIV-infected patients were tuberculosis (32.5%), candidiasis (29.3%), and PCP (2.4%), with PCP resulting in an in-hospital mortality rate of 33.1% [1]. The incidence of PCP has declined with combination of antiretroviral therapy (ART) and PCP prophylaxis. Nevertheless, PCP may occur in patients who are unaware of their HIV infection, are unable to reach medical care, or fail to adhere to PCP prophylaxis or ART [1, 2]. PCP can also progress rapidly into respiratory failure in patients not immediately treated with appropriate therapy. Early diagnosis of PCP in AIDS patients can be challenging, especially when the CD4 count is unknown; therefore, empiric therapy is often initiated based upon nonspecific clinical manifestations while awaiting the results of diagnostic tests [3].

Hematologic parameters obtained by automated hematologic analyzer including C-reactive protein (CRP), erythrocyte sedimentation rate (ESR), procalcitonin (PCT), white blood cell (WBC), lactate dehydrogenase (LDH), hydroxybutyrate dehydrogenase (HBDH), D-dimer, and Ferritin are rapid to test. CRP and LDH have been evaluated in the diagnosis of AIDS PCP and prediction of PCP severity [4, 5] but have been found to have low predictive value. Since cytokine responses differ for different types of infections, therefore specific patterns of cytokine response may have diagnostic and prognostic value [6]. Alveolar macrophages are the first line of host defense against* P. jirovecii*, leading to production of inflammatory chemokines IL-8 and IL-6 [7, 8]. Lung epithelial cells are the predominant cell type for* Pneumocystis* trophozoite and cyst adherence, which have been shown to produce IL-6, IL-8, and MCP-1 [9, 10]. Dendritic cells are also important effector immune cells in the lung; when exposed to* Pneumocystis *organisms, they displayed Th2 cytokine response, secreting IL-4 [11], and Th1-patterned cytokine response, secreting IL-1*β* and TNF-*α* [12]. In studies of mice infected with* P. jirovecii*, IFN-*γ* was found to augment host defense by increasing nitric oxide synthesis of the macrophages [13]. Therefore, potential biomarker panels including cell markers, cytokines, coagulation factors, acute phase proteins, and other immune molecules may be more predictive than a single biomarker. Previous studies of biomarkers for AIDS PCP evaluated only individual biomarker such as IL-8 and a few combinations [14–16]. In this study, we evaluated a large panel of biomarkers to determine which ones may be of value in predicting evolution of PCP in HIV-infected persons.

## 2. Materials and Methods

### 2.1. Study Population

**Figure 1:**
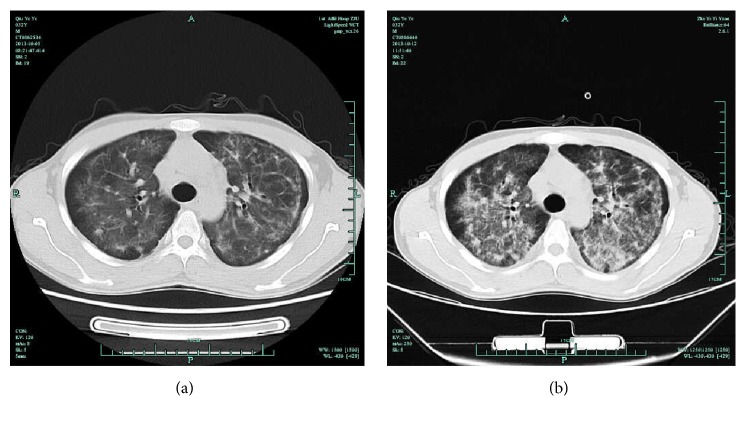
The chest images of computed tomography (CT) scan in the AIDS PCP patient. (a) shows bilateral, symmetric, interstitial, or granular opacities in typical case. (b) shows the opacities are perihilar and diffuse in severe case.

A total of 32 consecutive patients (>18 years of age) diagnosed with AIDS PCP at infectious department of The First Affiliated Hospital of Zhejiang University were enrolled from March 2013 to July 2014. HIV infection was confirmed as recommended by Center for Disease Control and Prevention (CDC). History of AIDS diagnosis was determined according to HIV/AIDS management guidelines. Among 32 subjects, three subjects had ART exposure but stopped ART by themselves 1 to 2 years ago prior to admission. Diagnosis of PCP was based on the combination of symptoms, signs, laboratory data, arterial oxygenation at rest, and computed tomography (CT), including subacute onset of unproductive cough, fever, progressive dyspnea, and chest discomfort that worsens within days to weeks; arterial partial pressure of oxygen (PaO_2_) lower than 65 mmHg at time admission; and elevated level of lactate dehydrogenase (>245 U/L). Typical chest CT imaging of AIDS PCP showed diffuse, bilateral, symmetrical interstitial infiltrates emanating from the hila in butterfly pattern (Figure 1) [3, 17].* Exclusion criteria *were as follows: (1) history of administration of agents with activity against PCP (including trimethoprim-sulfamethoxazole (SMZ-TMP), clindamycin, and caspofungin) or glucocorticoid therapy prior to enrollment; (2) evidence of other immune deficiencies (including malignancy, congenital immunodeficiency, and receipt of chemotherapy); and (3) evidence of immune reconstitution inflammatory syndrome (IRIS). We examined two clinical outcomes: severity of illness and survival; severity of illness was based on the worst oxygenation index during the hospitalization, defined as the ratio of PaO_2_/FiO_2_ (mmHg) and categorized into two groups: mild (PaO_2_/FiO_2_ > 200 mmHg) and severe (PaO_2_/FiO_2_ ≤ 200 mmHg). Subjects were also categorized into survival and nonsurvival groups based upon whether they survived the hospitalization.

Plasma was collected within the first 24 hours of admission for the measurement of targeted biomarkers. Written informed consents were obtained from all subjects. The study was conducted in accordance with the 1975 Declaration of Helsinki and approved by the Ethics Committee of The First Affiliated Hospital, School of Medicine, Zhejiang University, China.

### 2.2. Clinical Biochemical Parameters and Inflammatory Cytokines Examination

Biochemical parameters including WBC, eosinophil count, lymphocyte count, CRP, ESR, PCT, LDH, HBDH, D-dimer, and Ferritin were examined in a central laboratory of our hospital within 12 hours of admission. A 10 mL sample of peripheral blood was collected using the EDTA-anticoagulant tube. After centrifugation (1500 rpm for 5 minutes), the supernatant of blood was frozen and stored at −80° until it was analyzed. The levels of cytokines in the supernatant of blood were measured by the commercially enzyme-linked immunosorbent Human Platinum ELISA kits: IL-1*β*, IL-2, IL-4, IL-6, IL-10, IL-12, IL-17*α*, IL-18, MCP-1, and IFN-*γ* (eBioscience, North America).

### 2.3. Statistical Analysis

Data were expressed as mean ± SD. Kolmogorov-Smirnov tests were used to assess whether continuous variables are normally distributed, and then independent-samples *t*-test or nonparametric test (Mann-Whitney *U* test) was chosen to compare groups. Spearman rank correlation coefficient analysis was used for linear correlation analysis. Evaluation of severity performance of biomarkers (see Table 4) was analyzed using receiver operation characteristic (ROC) curves. The ROC curves were compared using a nonparametric method. Significance was defined as *P* < 0.05. Statistical analysis was performed using SPSS version 19 (SPSS, Armonk, New York, United States). All remaining statistical analyses were performed using MedCalc Statistical Software version 14.8.1 (MedCalc, Ostend, Belgium).

## 3. Results

### 3.1. Biochemical Makers in Severe Disease and Nonsurvivors

Clinical characteristics and baseline demographics of the 32 patients are shown in Table 1. Mean age was 40.72 ± 12.03 years, and mean CD4 count on admission was 87.14 ± 14.46/mm^3^. None of the patients had taken PCP prophylaxis prior to admission and 90.6% were naive to ART (three subjects had previous exposure but had not been on ART for at least 1 year prior to admission). HIV-RNA levels were higher in patients with PaO_2_/FiO_2_ ≤ 200 mmHg than in those with PaO_2_/FiO_2_ > 200 mmHg (Table 1).

**Figure 2:**
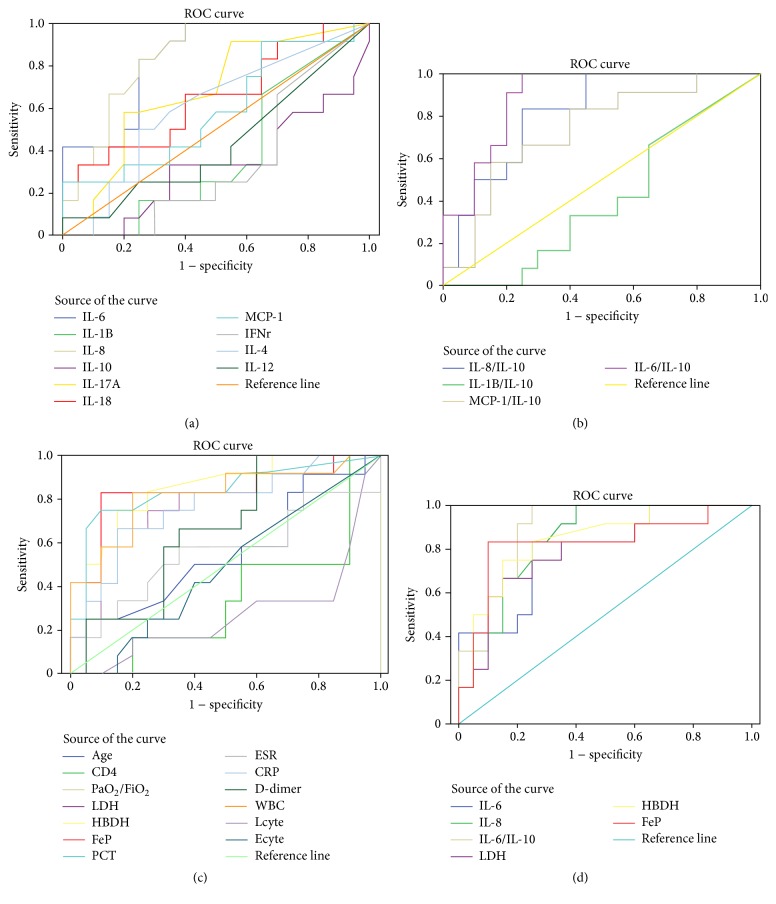
Receiver operating characteristic (ROC) curve analysis of biomarkers in lighter severity (PaO_2_/FiO_2_ ≤ 200 mmHg, *N* = 12) and heavier severity (PaO_2_/FiO_2_ > 200 mmHg, *N* = 20) AIDS PCP. (a) LDH (95% CI 0.636–0.956, *P* = 0.006), HBDH (95% CI 0.703–0.985, *P* = 0.001), and FeP (95% CI 0.657–0.993, *P* = 0.002) showing much higher AUC in severity AIDS PCP groups. (b) IL-6 (95% CI 0.704–0.976, *P* = 0.002) and IL-8 (95% CI 0.713–0.978, *P* = 0.001) showing much higher AUC in severity AIDS PCP groups. (c) IL-8/IL-10 (95% CI 0.671–0.962, *P* = 0.003), MCP-1/IL-10 (95% CI 0.559–0.916, *P* = 0.027), and IL-6/IL-10 (95% CI 0.781–1.000, *P* = 0.000) showing much higher AUC in severity AIDS PCP groups. (d) Much better six biomarkers' ROC curves shown together (WBC: white blood cell; LDH: lactate dehydrogenase; HBDH: hydroxybutyrate dehydrogenase; IL: interleukin; MCP-1: monocyte chemoattractant protein 1, and FeP: Ferritin protein).

**Figure 3:**
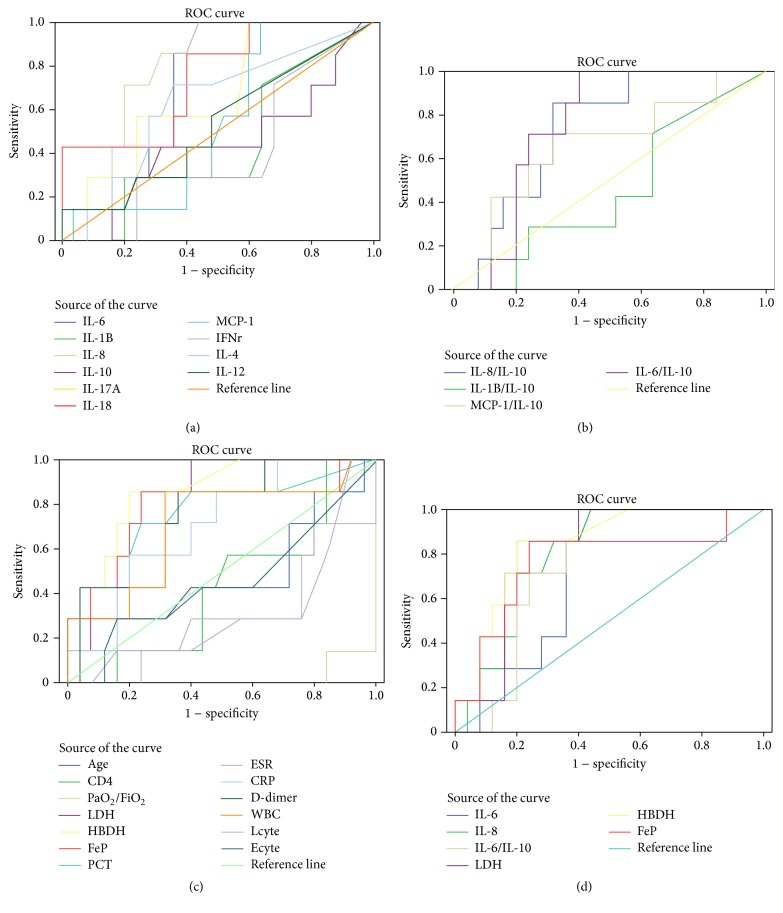
Receiver operating characteristic (ROC) curve analysis of biomarkers in nonsurvivors (*N* = 7) and survivors (*N* = 25) AIDS PCP. (a) LDH (95% CI 0.675–0.970, *P* = 0.010), HBDH (95% CI 0.797–0.989, *P* = 0.006), and FeP (95% CI 0.541–0.991, *P* = 0.034) showing much higher AUC in nonsurvivors AIDS PCP groups. (b) IL-6 (95% CI 0.547–0.898, *P* = 0.075) and IL-8 (95% CI 0.647–0.953, *P* = 0.017) showing much higher AUC in nonsurvivors AIDS PCP groups. (c) IL-8/IL-10 (95% CI 0.561–0.963, *P* = 0.057) and IL-6/IL-10 (95% CI 0.592–0.917, *P* = 0.043) showing much higher AUC in nonsurvivors AIDS PCP groups. (d) Much better six biomarkers' ROC curves shown together (WBC: white blood cell; LDH: lactate dehydrogenase; HBDH: hydroxybutyrate dehydrogenase; IL: interleukin; MCP-1: monocyte chemoattractant protein 1, and FeP: Ferritin protein).

**Figure 4:**
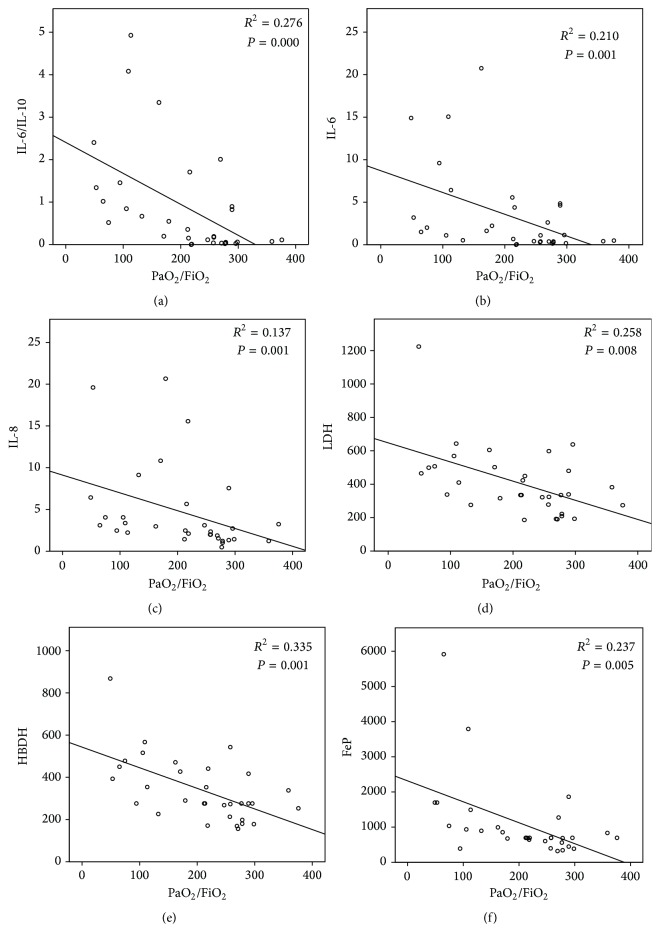
The correlation analysis of the oxygenation index with LDH, HBDH, FeP, IL-8, and IL-6 level and IL-6/IL-10 ratio. (a) IL-6/IL-10 (*P* = 0.000, *R* ^2^ = 0.276); (b) IL-6 (*P* = 0.001, *R* ^2^ = 0.210); (c) IL-8 (*P* = 0.001, *R* ^2^ = 0.137); (d) LDH (*P* = 0.008, *R* ^2^ = 0.258); (e) HBDH (*P* = 0.001, *R* ^2^ = 0.335); and (f) Ferritin (*P* = 0.005, *R* ^2^ = 0.237).

Blood levels of WBC, CRP, LDH, HBDH, and Ferritin were significantly higher in severe than in mild groups (Table 2). The levels of LDH, HBDH, and Ferritin were also markedly higher in nonsurvivors than in survivor groups (Table 3). In addition, ROC analysis of these biomarkers showed higher AUC in severity and nonsurvivors groups: LDH (95% CI 0.636–0.956, *P* = 0.006; 95% CI 0.675–0.970, *P* = 0.01), HBDH (95% CI 0.703–0.985, *P* = 0.001; 95% CI 0.697–0.989, *P* = 0.006), and Ferritin (95% CI 0.657–0.993, *P* = 0.002; 95% CI 0.541–0.991, *P* = 0.034) (Figures 2(a) and 3(a)). There were significant inverse correlations between the oxygenation index and plasma level of LDH (*P* = 0.008, *R* ^2^ = 0.258), HBDH (*P* = 0.001, *R* ^2^ = 0.335), and Ferritin (*P* = 0.005, *R* ^2^ = 0.237) (Figures 4(d), 4(e), and 4(f)).

### 3.2. Increased Levels of IL-8 and IL-6 in Severe Disease and Nonsurvivors

Plasma levels of IL-6 and IL-8 were significantly higher in patients with PaO_2_/FiO_2_ ≤ 200 mmHg than in those with PaO_2_/FiO_2_ > 200 mmHg (Table 3). Nonsurvivors also had significantly higher blood levels of IL-8 compared with survivors (Table 5). The ROC curves constructed are listed in Figures 2 and 3. Among tested cytokines, IL-6 and IL-8 had higher ROC in the severe and nonsurvival group. There were also significant inverse correlations between the oxygenation index and plasma levels of IL-6 (*P* = 0.001, *R* ^2^ = 0.210) and IL-8 (*P* = 0.001, *R* ^2^ = 0.137) (Figures 4(b) and 4(c)).

### 3.3. IL-6/IL-10 Ratio Was Intensively Related to Severe and Nonsurvival AIDS PCP Patients

The ratio between anti-inflammatory cytokines IL-10 and other proinflammatory cytokines IL-6, IL-8, IL-1*β*, and MCP-1 revealed higher levels both in the severe group and in nonsurvival groups (Tables 3 and 5). Severity and nonsurvivors groups had a higher ratio of IL-6/IL-10 (1.78 ± 1.56, *P* = 0.000; 1.11 ± 0.72, *P* = 0.043) (shown in Tables 3 and 5), higher AUC (95% CI 0.781–1.000, *P* = 0.000; 95% CI 0.592–0.917, *P* = 0.043) (Figures 2(c) and 3(c)), and more significantly inverse correlation with the oxygenation index (*P* = 0.01, *R* ^2^ = 0.276) (Figure 4(a)).

## 4. Discussion

Although LDH is a nonspecific biomarker and also increases in many other diseases, an elevated LDH level has been noted in many patients with PCP [18] and has been utilized for diagnosis of PCP [19–22]. Few articles or guidelines have assessed the utility of LDH in predicting severity of disease progression. In the present study, we observed significantly higher elevated levels of LDH in PCP patients developing severe respiratory compromise with PaO_2_/FiO_2_ ≤ 200 mmHg than those in those mild disease with PaO_2_/FiO_2_ > 200 mmHg. We also showed a significant inverse correlation between the oxygenation index and LDH (*P* = 0.008, *R* ^2^ = 0.258). The activity of HBDH is increased with PCP, correlating positively with LDH (*P* = 0.000, *R* ^2^ = 0.882, data not shown) and negatively with oxygenation index (*P* = 0.001, *R* ^2^ = 0.335), indicating that HBDH is likely to be reflection of the underlying lung inflammation. HBDH level seems to be another potential biochemical parameter for prediction of AIDS PCP severity and mortality.

Biochemical parameters such as CRP and D-dimer obtained via hematologic analyzer are rapid and require small quantities of blood samples. Similar to previous studies [5, 23–25], we found CRP and D-dimer to be useful parameters for the diagnosis and prediction of PCP severity. In our study, CRP levels were higher in patients with PaO_2_/FiO_2_ ≤ 200 mmHg, and D-dimer levels were higher in nonsurvivors.

Theoretically, abnormal exaggeration of proinflammatory or anti-inflammatory cytokine response can be harmful to the host, leading to respiratory distress syndrome, shock, multiorgan failure, or death. Therefore, quantitation of the inflammatory response may have prognostic implications [26]. The results of a previous study indicated that PCP patients had higher levels of proinflammatory cytokines compared to controls, but anti-inflammatory cytokines had a variable response. Some proinflammatory/anti-inflammatory ratios were valuable in assessing the severity of PCP and predicting the outcome of the patients [27].

IL-8 is correlated with both neutrophil infiltration of the lung and impaired gas exchange during severe PCP. Recent studies of PCP reported that the level of IL-8 in bronchoalveolar lavage fluid (BALF) and plasma may serve as a predictor of severe respiratory failure and death [15, 16]. Furthermore, plasma levels of IL-8 were found to be significantly higher in severe non-AIDS PCP [27]. In agreement with previous reports, the present study indicated that the blood level of IL-8 differed between subjects with PaO_2_/FiO_2_ ≤ 200 mmHg and PaO_2_/FiO_2_ > 200 mmHg, correlated inversely with oxygen index, and also differed between survivors and nonsurvivors [15].

IL-6 is a proinflammatory cytokine that is important for B cells as well as promoting CD4 T cell proliferation and survival [28]. In animal experiments, IL-6 levels were elevated in severe combined immunodeficient mice with* P. carinii* pneumonia compared with levels in* P. carinii*-free mice [29]. Kobayashi et al. [11] reported that BALF levels of IL-6 were higher in immunocompromised patients with PCP than in immunocompromised and immunocompetent patients without PCP [30]. In our study, plasma IL-6 levels were higher in patients with PaO_2_/FiO_2_ ≤ 200 mmHg than in those with PaO_2_/FiO_2_ > 200 mmHg but did differ between survivors and nonsurvivors. IL-10 is an anti-inflammatory cytokine produced primarily by antigen-presenting cells, which exerts negative regulatory effects on proinflammatory cytokines by downregulating their synthesis [31]. IL-10 is released in the lungs of experimental animals with PCP [32] and also in humans [33], resulting in decreased* Pneumocystis*-driven pulmonary inflammation and improved survival. In our study, IL-10 level in patients with PaO_2_/FiO_2_ > 200 mmHg were higher than that in PaO_2_/FiO_2_ ≤ 200 mmHg and similarly in survivors compared to nonsurvivors. Surprisingly, we found higher blood levels of IL-6/IL-10 ratio in patients with PaO_2_/FiO_2_ ≤ 200 mmHg and in nonsurvivors. Furthermore, IL-6/IL-10 had the highest AUC and correlated inversely with the oxygenation index (*P* < 0.001, *R* ^2^ = 0.276).

In conclusion, our data suggest that detection of plasma IL-8 levels and IL-6/IL-10 ratio combined with LDH and HBDH can predict the severity of PCP risk of death in AIDS patients with PCP. In developing countries where resources for mechanical ventilation are limited, assessment and risk stratification on admission, using commonly available laboratory biomarkers, may be particularly helpful for management of PCP.

## Figures and Tables

**Table 1:** Clinical characteristics and baseline demographics of AIDS PCP patients.

Characteristics	
Gender (M/F)	32 (32/0)
Age, years	40.72 ± 12.03
The most recent CD4 cell count	87.14 ± 14.46
HIV-RNA, copies/mL	
>200 mmHg, *N* = 14	23534.74 ± 23616.33
≤200 mmHg, *N* = 8	263789.65 ± 208749.16
PaO_2_/FiO_2_ before therapy	208.96 ± 90.03
>200 mmHg, *N* = 20	268.92 ± 44.26
≤200 mmHg, *N* = 12	109.00 ± 44.94
Mode of transmission	
Heterosexual transmission	19 (59.38%)
Homosexual transmission	12 (37.50%)
Bisexual transmission	1 (3.12%)
Underline disease	
Candidiasis (oral/pulmonary)	15 (10/5)
Syphilis (neurosyphilis)	6 (1)
Aspergillosis (esophagus/pulmonary)	6 (5/1)
Tuberculosis (intestinal/pulmonary)	2 (1/1)
Tinea cruris	1
Herpes zoster	1
CMV retinitis	1
Seborrheic dermatitis	1
Hematology disorders	1
Hepatic insufficiency	1
Psychonosema	1
Nutritional deficiencies	1
Hypokalemia	1
Pneumothorax	1
COPD	1
Hypertension	1
Treatment	
SMZ-TMP prophylaxis	0 (0%)
HARRT before PCP	3 (9.40%)
Anti-PCP therapy	32 (100%)
SMZ-TMP	32 (100%)
Clindamycin	32 (100%)
Caspofungin	11 (34.38%)
Antibiotic therapy	29 (90.63%)
Antifungal therapy	29 (90.63%)
Glucocorticoid therapy	30 (93.75%)
Mechanical ventilation	2 (6.25%)
Clinical outcome	
Survivor	25 (78.12%)
Nonsurvivor	7 (21.88%)

**Table 2:** Comparisons of laboratory data between PaO_2_/FiO_2_ ≤ 200 and >200 mmHg.

Laboratory data	Normal ranges	PaO_2_/FiO_2_ ≤ 200 mmHg, (*N* = 12)	PaO_2_/FiO_2_ > 200 mmHg, (*N* = 20)	*P* value
CRP	0.00–8.00 mg/L	75.73 ± 47.72	35.38 ± 35.97	0.011
ESR	0–15 mm/h	59.50 ± 32.03	55.00 ± 24.40	0.657
PCT	0.00–0.50 ng/mL	0.30 ± 0.32	0.05 ± 0.09	0.021
WBC	4.0–10.0 *∗* 10^9^/L	7.94 ± 2.89	4.96 ± 1.67	0.005
Eosinophil count	0.20–0.50 *∗* 10^9^/L	0.07 ± 0.12	0.11 ± 0.20	0.871
Lymphocyte count	0.8–4.0 *∗* 10^9^/L	0.63 ± 0.33	0.88 ± 0.39	0.071
LDH	109–245 U/L	529.50 ± 246.73	335.20 ± 130.73	0.007
HBDH	72–182 U/L	443.00 ± 168.54	276.55 ± 101.18	0.001
D-dimer	0–700 ug/L	2904.17 ± 3668.19	1995.06 ± 3732.31	0.129
Ferritin	7.0–323.0 ng/mL	1698.33 ± 1590.71	696.12 ± 344.71	0.002

Data are expressed as mean and SD. CRP: C-reactive protein; ESR: erythrocyte sedimentation rate; PCT: procalcitonin; WBC: white blood cell; LDH: lactate dehydrogenase; HBDH: hydroxybutyrate dehydrogenase.

**Table 3:** Comparisons of cytokines between PaO_2_/FiO_2_ ≤ 200 and >200 mmHg.

Cytokines	PaO_2_/FiO_2_ ≤ 200 mmHg, (*N* = 12)	PaO_2_/FiO_2_ > 200 mmHg, (*N* = 20)	*P* value
IL-6, pg/mL	6.58 ± 6.87	1.42 ± 1.86	0.002
IL-1*β*, pg/mL	1.11 ± 2.04	3.62 ± 6.56	0.178
IL-8, pg/mL	7.41 ± 6.52	3.01 ± 3.38	0.001
IL-10, pg/mL	3.85 ± 2.50	7.73 ± 9.40	0.175
IL-17*α*, pg/mL	0.34 ± 0.28	0.18 ± 0.25	0.102
IL-18, pg/mL	588.52 ± 463.57	334.16 ± 176.31	0.091
MCP-1, pg/mL	198.85 ± 160.17	143.28 ± 99.03	0.233
IFN*γ*, pg/mL	0.56 ± 1.02	2.95 ± 5.56	0.128
IL-4, pg/mL	1.17 ± 1.61	1.43 ± 3.24	0.314
IL-12, pg/mL	0.24 ± 0.40	0.32 ± 0.39	0.585
IL-8/IL-10	3.08 ± 3.52	0.96 ± 1.86	0.003
IL-1*β*/IL-10	0.20 ± 0.30	0.76 ± 1.48	0.210
MCP-1/IL-10	75.26 ± 104.70	33.75 ± 36.66	0.027
IL-6/IL-10	1.78 ± 1.56	0.34 ± 0.58	0.000

IL: interleukin; MCP1: monocyte chemoattractant protein 1; IFN*γ*: interferon*γ*.

**Table 4:** Comparisons of biomarkers between nonsurvivors and survivors of AIDS PCP patients.

Laboratory data	Nonsurvivors (*N* = 7)	Survivors (*N* = 25)	*P* value
CRP, mg/L	73.16 ± 49.78	44.17 ± 42.02	0.131
ESR, mm/h	41.57 ± 27.21	60.92 ± 26.03	0.095
PCT, ng/mL	0.21 ± 0.22	0.13 ± 0.24	0.080
WBC, *∗*10^9^/L	7.73 ± 3.44	5.61 ± 2.20	0.057
Eosinophil	0.09 ± 0.15	0.09 ± 0.18	0.703
Lymphocyte	0.61 ± 0.33	0.83 ± 0.39	0.194
LDH, U/L	586.29 ± 289.93	358.16 ± 142.67	0.006
HBDH, U/L	486.86 ± 184.58	297.56 ± 114.45	0.002
D-dimer, ug/L	4286.86 ± 4365.60	1789.72 ± 3356.78	0.047
Ferritin, ng/mL	1789.83 ± 1878.50	870.95 ± 702.22	0.033

SD, AIDS, PCP, CRP, ESR, PCT, WBC, LDH, and HBDH as shown in Tables 1 and 2.

**Table 5:** Comparisons of cytokines between nonsurvivors and survivors of AIDS PCP patients.

Cytokines	Nonsurvivors (*N* = 7)	Survivors (*N* = 25)	*P* value
IL-6, pg/mL	4.85 ± 5.32	2.93 ± 4.98	0.075
IL-1*β*, pg/mL	1.55 ± 2.57	2.99 ± 5.55	0.609
IL-8, pg/mL	7.22 ± 6.51	3.95 ± 4.76	0.017
IL-10, pg/mL	4.31 ± 2.77	6.83 ± 8.62	0.632
IL-17*α*, pg/mL	0.39 ± 0.33	0.20 ± 0.24	0.109
IL-18, pg/mL	749.28 ± 540.36	340.02 ± 179.33	0.091
MCP-1, pg/mL	164.16 ± 126.94	164.11 ± 128.29	0.093
IFN*γ*, pg/mL	0.78 ± 1.28	2.41 ± 5.07	0.431
IL-4, pg/mL	1.36 ± 1.81	1.32 ± 2.95	0.279
IL-12, pg/mL	0.30 ± 0.47	0.28 ± 0.38	0.826
IL-8/IL-10	2.45 ± 2.69	1.56 ± 2.8	0.059
IL-1*β*/IL-10	0.23 ± 0.36	0.64 ± 1.32	0.658
MCP1/IL-10	49.12 ± 26.97	49.37 ± 80.28	0.210
IL-6/IL-10	1.11 ± 0.72	0.82 ± 1.37	0.043

SD, IL, MCP1, and IFN*γ* as shown in Tables 1 and 2.
